# Factors associated with patients’ satisfaction in Brazilian dental primary health care

**DOI:** 10.1371/journal.pone.0187993

**Published:** 2017-11-16

**Authors:** Muath Abdullah Aldosari, Mary Angela Tavares, Antônio Thomaz Gonzaga Matta-Machado, Mauro Henrique Nogueira Guimarães Abreu

**Affiliations:** 1 Department of Oral Health Policy and Epidemiology, Harvard School of Dental Medicine, Boston, MA, United States of America; 2 Department of Periodontics and Community Dentistry, College of Dentistry, King Saud University, Riyadh, Kingdom of Saudi Arabia; 3 Senior Clinical Investigator, The Forsyth Institute, Cambridge, Massachusetts, United States of America; 4 Programme Director, Dental Public Health, Department of Oral Health Policy and Epidemiology, Harvard School of Dental Medicine, Boston, Massachusetts, United States of America; 5 Associate Professor, Department of Social and Preventive Medicine, Universidade Federal de Minas Gerais, Belo Horizonte, Brazil; 6 Associate Professor, Department of Community and Preventive Dentistry, Universidade Federal de Minas Gerais, Belo Horizonte, Brazil; University of West London, UNITED KINGDOM

## Abstract

**Objective:**

To assess factors associated with patients’ satisfaction with the treatment by dentists in primary health care (PHC) in Brazil.

**Materials and methods:**

The dataset was part of a nationwide cross-sectional survey for evaluating PHC teams conducted by the Brazilian Ministry of Health. Patients from each of 16,202 oral health teams were interviewed. In addition to sociodemographic information, the questionnaire included information about patient experience domains: access and booking of dental appointments, bonding and accountability, welcoming of the patient, and their perception of dental facilities.

**Statistical analysis:**

The dependent variable was the answer to the question ‘From 0 to 10, how would you grade your satisfaction with treatment received from the dentist?’ Negative binomial regression models were used to estimate the unadjusted and adjusted rate ratios and corresponding 95% confidence interval.

**Results:**

The mean patient satisfaction was 9.4 (±2.3). Higher patient satisfaction with PHC was associated with lower education and the patient’s perception of the clinic conditions. Moreover, higher satisfaction was associated with positive reception and hospitality, enough time for treatment, and instructions that met patients’ needs. Lower satisfaction with PHC was associated with patients who have jobs compared to those who do not work.

**Conclusion:**

Patient satisfaction is increased with friendly and understanding PHC staff. Moreover, meeting patient expectations by taking time to understand the needs and giving the right instructions is associated with higher satisfaction.

## Introduction

Since the declaration of Alma-Ata in September 1978, primary health care (PHC) has been considered a key to achieve the goal of ‘health for all’ [1]. The comprehensiveness and integrated nature of PHC have been associated with better health outcomes [2] and fewer visits to specialists or emergency departments [3, 4]. In addition, PHC serves as a regular entry point that brings health care closer to the people, which improves access to care and reduces social injustice [5–7]. This will ultimately improve patient satisfaction [8] and lead to better compliance and efficient use of resources [2, 6, 9].

Satisfaction of the primary care users is one indicator of health care quality. Donabedian proposes that health services could be evaluated according to structure, work process, results, and user satisfaction [10]. Satisfaction is a patient-based outcome measure. Patients are the end consumer of the PHC, so they can identify areas for improvement and address users’ needs, like waiting time and staff communication. According to the theoretical model of Andersen and Davidson, the dental health system and personal characteristics affect patients’ behaviour, which, in turn, determines the user satisfaction with dental services [11]. Higher satisfaction also has been linked to measures of health outcomes by compliance with treatment and keeping up with appointments [12]. Moreover, a patient’s level of satisfaction can be considered an expression of his or her expectations in relation to his or her actual experiences [13].

Primary care is the basic unit of the Brazilian health care system Unified Brazilian Health System (‘Sistema Único de Saúde’ [SUS]). SUS is the largest federally funded universal system in the world, providing health care to more than 200 million people [14]. In 2004, the national oral health initiative, Smiling Brazing (‘Brasil Sorridente’), was recognised as one of the four priority areas of the SUS. Oral primary care serves as its backbone and the entry level of the patient to Smiling Brazil. It is expected to solve 80% of patients’ problems, after which patients are referred to more specialised care [15]. However, the ambitious oral primary care in Smiling Brazil faces many challenges, including but not limited to staffing shortage, excess demand, and primary-to-secondary care integration [16, 17].

Although the interest in patient satisfaction is still growing, the concept remains challenging to define theoretically [12]. Developing a conceptual model for patient satisfaction is difficult [18]. A recent systematic review in 2016 demonstrated that patient satisfaction determinants vary widely between studies and are not conclusive [19]. Also, empirical evidence has demonstrated a multidimensional concept of patient satisfaction [20]. Understanding the factors associated with reported patient satisfiers or dissatisfiers would help in defining and measuring the satisfaction with health services. Moreover, factors of patient satisfaction are highly culture-dependent [21–23].

In this paper, the data were collected from a national survey to better understand the factors associated with Brazilian patient satisfaction in primary dental settings. This study evaluated three different dimensions of satisfaction: demographic characteristics, patient experience, and their perception toward the dental facilities. The aim of the study is to assess the factors associated with patient satisfaction with dental treatment by exploring five domains: socio-demographic characteristics, access and scheduling of dental appointments, welcoming the patient, bonding with the patient, and patient’s perception of the clinic conditions.

## Materials and methods

### Ethics statement

This study was submitted to and approved by the Brazilian Ethics Committee and by the Ethics Committee for Human Research of the Universidade Federal de Minas Gerais (protocol no. CAAE 02396512.8.0000.5149). Patients signed an informed consent form; the participation was voluntary, and patients could refuse to answer any of the questions. Publicly available, de-identified data from the Brazilian Ministry of Health were analysed.

### Population

The second cycle of a programme to assess and enhance the quality of PHC, the National Programme for Improving Access and Quality of Primary Care (‘*Programa Nacional de Melhoria do Acesso e da Qualidade da Atenção Básica*’ [PMAQ-AB]), was evaluated. This cross-sectional survey was based on the classic quality of care framework by Donabedian, in which quality is evaluated using structure, process, and outcome parameters [10]. Patients who were at PHC settings at any time during the external evaluation of the PMAQ were invited to participate in this structured interview. At least four patients who were waiting to receive health treatment, for each PHC, were interviewed. No random selection technique was used. Out of 114,615 patients interviewed at 23,934 PHC centres in Brazil, 37,262 answered questions about satisfaction with dental treatment at 16,107 PHC centres in which patients had been previously treated by dentists. Only patients who answered all the questions were included in this study. No information on the rate of patient refusal in answering this interview is available.

### Data collection

Data were collected from November 2013 to April 2014. The structured questionnaire was developed through a partnership between the Brazilian Ministry of Health and a committee of six academic institutions throughout the country. The face-to-face interviews were conducted by trained researchers, all with higher education in the health area. A pilot study was performed prior to the fieldwork. The survey was conducted by using computer tablets through a programme designed specifically for the PMAQ-AB. The Ministry of Health assessed participation by telephone. All interviews were carried out at the PHC setting. In Brazil, PHC settings included health teams composed by physicians, nurses, dentists, and health assistants. These professionals work together at the same building, and each team is responsible for the health of around 2,400 to 4,000 individuals.

The items of the questionnaire involved sociodemographic information in addition to the four domains of interest: access and booking of dental appointments, bonding and accountability, welcoming of the patient, and their perception of dental facilities. Most of the questions have either ordinal or dichotomous answers. In addition, each question included an option for ‘no answer/do not know’. Missing data were not computed in statistical analysis.

### Variables used for analysis

The dependent variable analysed was the question ‘From 0 to 10, which grade do you consider for your satisfaction with treatment received by the dentist?’ Higher values reflect better satisfaction with the dentist’s treatment. The minimum, mean, median, and maximum values of patients’ satisfaction were calculated for each covariate.

Negative binomial regression models were used to estimate the unadjusted and adjusted rate ratios (RR) and corresponding 95% confidence interval. Firstly, we carried out unadjusted negative binomial regression models to estimate unadjusted RR (CI95%) and p values for each of 23 covariates separately. In this first step, any covariate with a p value less than 0.25 was a candidate to be tested in the final adjusted negative binomial regression model. Because the interest was focused on the independent effects of each covariate, all potential variables were included in the unadjusted model, which included sex, age, education level (nine categories), working status (binary), nine questions about the access and booking of dental appointments, three questions about bonding and accountability, three questions about welcoming of the patients, and four questions about the patient’s evaluation of the health service (S1 Questionnaire). Only covariates with a p value less than 0.05 were maintained in the final model. For the evaluation of goodness of fit of the final model, the ratio between residual deviance and degree of freedom and the chi-squared test of the residual deviance results was indicated [24, 25].

Two secondary analyses were carried out with a cut-off point for patient satisfaction on percentile 10 (satisfaction level from zero to eight versus grades 9 and 10) and another on percentile 25 (satisfaction level from zero to nine versus satisfaction grade 10). For the latter, two separated binary logistic regression models were developed using Forward Stepwise Wald strategies. All analyses were developed using SPSS version 18.0 (SPSS, Chicago, IL, USA).

## Results

The surveyed sample of 9,120 patients was compared with those excluded from the analysis (n = 28,142) using the four sociodemographic variables. The parameters for all variables were similar. The mean age (DP) was 38.7 (12.2) and 40.1 (15.2) for individuals included (n = 9,120) and excluded (n = 28,142) from the regression, respectively; 83.9% were female (n = 9,120) versus 82.1% of females in the other group (n = 28,142); 61.4% did not work at group included in this survey (n = 9,120) versus 64.1% among excluded patients (n = 28,142). Schooling was also similar; for example, 34.0% and 34.7% had one to seven years of education, respectively, from individuals included and excluded from the analysis. The descriptive characteristics of patients included in this study are presented in Table 1.

**Table 1:** Characteristics of the patients interviewed in primary health care, Brazil, 2013–2014.

***Quantitative variables (N = 9*,*120)***	**Mean (SD), Median**	**Minimum–Maximum**
**Age (years)**	38.7 (14.2); 36	16–97
**From 0 to 10, which grade do you consider for your satisfaction with the treatment received by the dentist?**	9.4 (±1.2); 10	0–10
***Categorical variables (N = 9*,*120)***	**Frequency**	**%**
**Female sex**	7,656	83.9
**Education level**		
Illiterate	324	3.5
Read and write	346	3.8
From 1 to 7 years of education	3,100	34.0
8 years of education	1,025	11.2
9 to 10 years of education	1,147	12.6
11 years of education	2,497	27.4
Incomplete college	317	3.5
Graduated college	281	3.1
Post-graduate	83	0.9
**Do you work? (Yes)**	3,523	38.6
**Most of the time, do you make your appointment with the dentist by phone call? (Yes)**	509	5.6
**Most of the time, do you make your appointment with the dentist using the internet? (Yes)**	11	0.1
**Most of the time, do you make your appointment personally visiting the PHC? (Yes)**	6,215	68.1
**Most of the time, do you make an appointment by filling out a formal paper? (Yes)**	1,762	19.3
**Most of the time, do you need to get in line to fill out formal papers to make the appointment? (Yes)**	1,491	16.2
**Most of the time, do you make your appointment with the dentist in the PHC by the community health agent? (Yes)**	1,717	18.8
**When given an appointment with the dentist, your appointment is:**		
Other way	35	0.4
At a specific period of the day	258	2.8
In order of arrival	5,721	62.7
Trying to fit you, with no guarantee	51	0.6
At a specific time	3,055	33.5
**Have you ever left the dental clinic with the next appointment scheduled? (Yes)**	6,703	73.5
**Waiting time for dental appointment (up to 7 days)**	5,847	64.1
**In the clinic, how often were you guided by the oral health professionals about your health?**		
Always	7,409	81.2
Almost always	1,281	14.1
Almost never	228	2.5
Never	202	2.2
**During dental treatment, do the oral health professionals take notes in your dental records?**		
Yes, always	8,701	95.4
Yes, sometimes	248	2.7
No	171	1.9
**Do you think the time for dental treatment is enough?**		
Yes, always	8,139	89.2
Yes, sometimes	706	7.8
No	275	3.0
**When you looked for dental care without an appointment, did you receive care?**		
Yes, always	6,566	72.0
Sometimes/No	2,554	28.0
**What do you think about the way you were treated (or welcomed) when entering the oral health service?**		
Very good	3,113	31.5
Good	5,359	58.4
Reasonable	591	8.9
Bad	41	0.8
Very bad	16	0.3
**Does the oral health information given to you in the clinic meet your needs?**		
Yes, always	7,748	85.0
Yes, sometimes	1,142	12.5
No	230	2.5
**In general, do you think the facilities of the dental office are in good clean condition? (Yes)**	8,768	96.1
**In general, do you think the facilities of the dental office have good ventilation or air conditioning? (Yes)**	8,342	91.5
**In general, do you think the dental equipment is in good working condition? (Yes)**	8,156	89.4
**In general, do you think the dental chair is in good working condition? (Yes)**	7,732	84.8

Brazilian patients’ satisfaction with dentists in PHC varied from 0 to 10, with an average of 9.4 (±1.2) (Table 1). The final multivariate adjusted model (N = 9,120 patients with no missing data) comprised 10 variables.

Male patients were less likely to be satisfied with dental treatment compared to women (RR = 0.99; CI95% = 0.99–1.00). The decrease in education level showed an increase in patient satisfaction with PHC. Working patients reported less satisfaction with PHC compared to those who do not work (RR = 0.99; CI95% = 0.99–1.00).

The frequency that patients were guided by the oral health professionals about health care is associated with patient satisfaction; patients who received an appointment in order of arrival are more likely to be satisfied compared with those who were treated at a specific time; those who considered that the time for dental treatment was enough; the patient’s positive perception about the way that they were treated (or welcomed) when entering the oral health service; those who considered that the oral health information given to them in the clinic met their needs; and the good perception about the cleanliness of the dental office and that the dental chair was in good working condition were more prone to be satisfied with the dentist (Table 2). Parameters of goodness of fit were adequate. Chi-squared test of the residual deviance results with a p value equal to 1, indicating that the model fit well. The secondary analyses showed similar associations between patient satisfaction and covariates (S1 and S2 Tables).

**Table 2:** Factors associated with patient satisfaction with dentists (n = 9,120) in primary health care, Brazil, 2013–2014.

Variable	Satisfaction Scores (mean; median)	Unadjusted Rate Ratio (CI 95%)	P value	Adjusted Rate Ratio (CI 95%)	P Value
**1. Demographic Characteristics**					
**Sex**					
Male	9.32; 10	1.00 (0.99–1.00)	0.397	0.99 (0.99–1.00)	0.029
Female	9.35; 10	1		1	
**Age**			<0.001		
**Education level**					
Post-graduate	9.27; 9	0.96 (0.94–0.98)	0.001	0.97 (0.95–0.99)	0.005
Graduated college	9.13; 9	0.95 (0.93–0.97)	<0.001	0.95 (0.93–0.97)	<0.001
Incomplete college	9.20; 10	0.95 (0.94–0.97)	<0.001	0.96 (0.95–0.98)	<0.001
11 years of education	9.24; 10	0.96 (0.95–0.97)	<0.001	0.97 (0.95–0.98)	<0.001
9 to 10 years of education	9.29; 10	0.96 (0.95–0.98)	<0.001	0.97 (0.96–0.99)	0.001
8 years of education	9.33; 10	0.97 (0.95–0.98)	<0.001	0.97 (0.96–0.99)	0.002
From 1 to 7 years of education	9.44; 10	0.98 (0.96–0.99)	0.002	0.98 (0.97–1.00)	0.039
Read and write	9.54; 10	0.99 (0.97–1.01)	0.240	0.99 (0.97–1.01)	0.287
Illiterate	9.65; 10	1		1	
**Do you work?**					
Yes	9.27; 10	0.99 (0.98–0.99)	<0.001	0.99 (0.99–1.00)	0.001
No	9.40; 10	1		1	
**2. Access and Booking of Dental Appointments**					
**Most of the time, do you make your appointment with the dentist by phone call?**					
Yes	9.35; 10	1.00 (0.99–1.01)	0.992		
No	9.35; 10	1			
**Most of the time, do you make your appointment with the dentist using the internet?**					
Yes	9.45; 10	1.01 (0.96–1.07)	0.685		
No	9.35; 10	1			
**Most of the time, do you make your appointment personally visiting the PHC?**					
Yes	9.36; 10	1.00 (1.00–1.01)	0.148		
No	9.32; 10	1			
**Most of the time, do you make an appointment by filling out a formal paper?**					
Yes	9.33; 10	1.00 (0.99–1.00)	0.440		
No	9.35; 10	1			
**Most of the time, do you need to get in line to fill out formal papers to make the appointment?**					
Yes	9.25; 10	0.99 (0.98–1.00)	0.003		
No	9.36; 10	1			
**Most of the time, do you make your appointment with the dentist in the PHC by the community health agent?**					
Yes	9.44; 10	1.01 (1.01–1.02)	<0.001		
No	9.32; 10	1			
**When given an appointment with the dentist, your appointment is:**					
Other way	9.14; 10	0.98 (0.93–1.03)	0.404	1.00 (0.95–1.04)	0.851
At a specific period of the day	9.07; 10	0.97 (0.95–0.99)	0.014	0.99 (0.97–1.02)	0.556
In order of arrival	9.33; 10	1.00 (1.00–1.01)	0.244	1.01 (1.00–1.02)	0.001
Trying to fit you, with no guarantee	9.39; 10	1.01 (0.98–1.04)	0.689	1.02 (0.99–1.05)	0.190
At a specific time	9.33; 10	1		1	
**Have you ever left the dental clinic with the next appointment scheduled?**					
Yes	9.41; 10	1.03 (1.02–1.03)	<0.001		
No	9.18; 10	1			
**Waiting time for dental appointment**					
Up to 7 days	9.43; 10	1.03 (1.02–1.03)	<0.001		
8 days or more	9.20; 10	1			
**3. Bonding and Accountability**					
**In the clinic, how often were you guided by the oral health professionals about your health?**					
Never	8.16; 9	0.86 (0.83–0.89)	<0.001	0.96 (0.93–1.00)	0.028
Almost never	7.80; 8	0.82 (0.79–0.86)	<0.001	0.91 (0.88–0.95)	<0.001
Almost always	8.88; 9	0.94 (0.93–0.94)	<0.001	0.97 (0.97–0.98)	<0.001
Always	9.51; 10	1		1	
**During dental treatment, do the oral health professionals take notes in your dental records?**					
No	8.63; 9	0.92 (0.89–0.95)	<0.001		
Yes, sometimes	8.52; 9	0.91 (0.88–0.93)	<0.001		
Yes, always	9.38; 10	1			
**Do you think the time for dental treatment is enough?**					
No	7.55; 10	0.80 (0.76–0.83)	<0.001	0.88 (0.85–0.91)	<0.001
Yes, sometimes	8.43; 10	0.89 (0.88–0.90)	<0.001	0.95 (0.93–0.96)	<0.001
Yes, always	9.49; 10	1		1	
**4. Welcoming of the Patient**					
**When you looked for dental care without an appointment, did you receive care?**					
Yes, always	9.47; 10	1.05 (1.04–1.06)	<0.001		
Sometimes/No	9.04; 10	1			
**What do you think about the way you were treated (or welcomed) when entering the oral health service?**					
Very bad	6.06; 8	0.62 (0.49–0.79)	<0.001	0.69 (0.55–0.87)	0.002
Bad	6.72; 7	0.69 (0.62–0.77)	<0.001	0.77 (0.70–0.86)	<0.001
Reasonable	8.11; 8	0.83 (0.82–0.84)	<0.001	0.89 (0.87–0.90)	<0.001
Good	9.37; 10	0.96 (0.96–0.96)	<0.001	0.97 (0.97–0.98)	<0.001
Very good	9.76; 10	1		1	
**Does the oral health information given to you in the clinic meet your needs?**					
No	7.12; 8	0.75 (0.71–0.78)	<0.001	0.86 (0.82–0.90)	<0.001
Yes, sometimes	8.55; 9	0.90 (0.89–0.91)	<0.001	0.96 (0.95–0.97)	<0.001
Yes, always	9.53; 10	1		1	
**5. Perception of Dental Facilities**					
**In general, do you think the facilities of the dental office are in good clean condition?**					
No	8.49; 10	0.91 (0.88–0.93)	<0.001	0.97 (0.94–0.99)	0.001
Yes	9.38; 10	1		1	
**In general, do you think the facilities of the dental office have good ventilation or air conditioning?**					
No	8.85; 10	0.94 (0.93–0.96)	<0.001		
Yes	9.39; 10	1			
**In general, do you think the dental equipment is in good working condition?**					
No	8.85; 10	0.94 (0.93–0.95)	<0.001		
Yes	9.40; 10	1			
**In general, do you think the dental chair is in good working condition?**					
No	9.08; 10	0.97 (0.96–0.98)	<0.001	0.99 (0.98–0.99)	<0.001
Yes	9.39; 10	1		1	

## Discussion

Ten factors were associated with Brazilian patient satisfaction: three demographic characteristics and two factors in each of three domains and one factor in the access and booking domain. Male, working patients and patients with a higher education level were less likely to be satisfied with dentists in PHC. When patients perceived some aspects of bonding and welcoming in PHC in a positive way, they were more likely to be satisfied with the dental treatment. Patients who had a good perception about the structure of the dental office at the PHC centre were more prone to be satisfied with the dentist. Finally, patients who received an appointment in order of arrival were more likely to be satisfied compared with those who were treated at a specific time.

Most PHC users in our sample were females (83.9%) or non-workers (61.4%), which could reflect a high number of housewives. Among the demographic characteristics, education level was found to have the strongest association with patient satisfaction. There was an inverse relationship with lower-educated patients reporting the highest satisfaction. A similar trend was found in different cultural and political settings [26–28]. Moreover, gender and working status of the patients have a similar strength of association with satisfaction. Males were slightly less likely to be satisfied with dental health services. This result corroborates the gender experience in Brazilian PHC [29]. Similar findings also were documented in different health systems around the world [23, 26, 30]. In contrast to Brazilian primary medical services, working patients reported lower satisfaction in dental care compared to non-workers. One explanation of these associations is that highly educated and working patients could have greater access to more available and flexible private dental care. Another explanation could be the increased awareness about autonomy among highly educated patients, challenging the paternalistic decision-making practiced by the majority of health care providers [31]. However, demographic characteristics in general are considered inconsistent in the literature and weakly associated with the satisfaction with health care [19]. This inconsistency could reflect the nature of satisfaction in different cultures. Moreover, these associations could be considered small, and any proposal of translating this information into policy actions should be viewed with caution.

The only factor associated with satisfaction from the booking and access domain was the way the appointment with the dentist was made. Patients who reported that their appointment was made in order of arrival were slightly more prone to be satisfied with the dentist. It is not an easy task to explain this result. There is some evidence that a first-come-first-serve basis is well evaluated by patients [32] or not [33]. In Brazil, a first-come-first-serve basis is not the recommended approach for organising patients´ appointments, because those with more severe oral conditions may not be prioritised. However, it appears that patients are slightly more prone to be satisfied with the dentist with this approach. This result requires a deepened scientific approach, such as qualitative methods, to understand the feelings and behaviours of individuals under dental treatment in PHC.

The welcoming of patients when entering primary dental care was the most significant factor associated with satisfaction in our study. The relationship between the patient´s perception of welcoming and satisfaction had a ‘dose-response gradient’. As this perception worsens, the satisfaction also worsens. Interpersonal care factors like friendliness, caring, and sympathy are the most reported determinants associated with satisfaction in the health services literature [19]. Bonding with the patient by thoughtfully asking about his or her health beyond issues related to the chief complaint was associated with higher satisfaction. This simple method has been shown to boost patient satisfaction in both Brazilian dental and medical settings [27]. In addition, giving the patients enough time to express their concerns will open a two-way communication that makes patients feel heard and part of the decision-making process. It will not only lead to higher satisfaction but also help the dental staff to meet the patients’ needs and expectations [34]. Reported sufficient time and meeting needs were associated with higher satisfaction in our sample, as well as in the medical sample [27]. Therefore, the organisation and management of PHC should seriously consider that the welcoming and bonding dimensions are key factors of the quality of health services.

Patients’ perception towards the physical environment in our sample had a positive association with the experience and the overall satisfaction. Our findings are supported by the dental literature. Kikwilu et al. reported that Tanzanian patients’ satisfaction was associated with the cleanliness of the dental clinic [21]. A worse perception about the physical environment and cleanliness of the dental office may result in a negative view of PHC, with dissatisfaction as a consequence [35]. Both parameters that influenced patient satisfaction are essential structures of PHC. Although patients´ perceptions about cleanliness were not accurate in terms of the risk of infection, the only benefit from a visual inspection is to appease aesthetic obligations [36]. Furthermore, a dental chair in good working condition is, at least, essential for dental care in PHC.

The following variables were tested for association with patient satisfaction: age, different ways of booking an appointment, leaving the clinic with the next appointment scheduled, waiting time for a dental appointment, taking notes in the patient’s record, the ability to receive emergency care with no appointment, and two variables about the perception of dental facilities. These variables were collected as part of the national PMAQ-AB efforts to evaluate users’ experience in the health system. However, none of them were associated with our outcome.

Factors that do not seem to affect satisfaction among the users of PHC in Brazil are making an appointment in person and the need to fill out formal papers. This could be because patients perceived these factors as the norm, not an inconvenience. Only 11 patients booked their appointments using the internet. This under-utilisation could be attributed to a combination of a lack of promotion and a low-quality electronic portal, or it could reflect the actual internet usage among the PHC patients. Moreover, patients seem indifferent regarding the timing of appointment schedules. Such indifference could reflect the patients’ expectation of their health care unit. There is also a curious lack of association between waiting time and satisfaction. Such delayed access to health services, even for non-communicable chronic conditions, has been associated with negative consequences and is documented to lower user satisfaction [37–40]. However, this association was not identified in the present survey.

A major limitation of this study is the high level of reported satisfaction and lack of variation. This problem in measuring satisfaction is well reported in most of the literature on health services [18]. Positively skewed reporting could be attributed to social desirability bias, unwillingness to express negative opinions about providers, or a lack of clearance and specificity of questions [41–44]. However, Fox and Storms [45] argued that the lack of variability makes expressed factors of dissatisfaction highly valuable. Williams [46] suggested that the expression of dissatisfaction is a sign of an ‘extremely bad’ factor. In addition to the methodological difficulties in measuring satisfaction, visit-specific factors were not collected, such as the type of procedure (preventive vs restorative), and pain experience could affect the patient’s experience and satisfaction. Lastly, only the patients who answered all the questions were included in the final analysis. Despite the similarity between patients included and excluded from our analysis, it is hard to assume a non-differential response, which might affect the inferences.

SUS is a unique health care system, as it is the largest established model of a single-payer system in the world. The federally funded Smiling Brazil, the oral care component of SUS, serves more than 200 million people, 85.7% of whom live in urban areas [47]. Our understanding of the challenges that SUS faces would help us to shape a better model of governmental health services that is just and guarantees equal access to health care. One of the most important consequences of the PMAQ-AB survey results is that government funds are based on these indicators. The patient satisfaction component carries a low weight of 10% in the final evaluation, but it is crucial to assess the validity of this component.

Within the limitations of this study, satisfaction among Brazilian patients who use dental PHC is affected mainly by the first impression when welcomed upon entering the clinic. Moreover, caring for the patient’s health, taking the time to understand the patient’s needs, and addressing them improves their satisfaction. In addition to interpersonal skills, a combination of physical environment and demographic factors plays a role in patients’ ultimate satisfaction. The results of the present survey provide an opportunity for organisation managers and policy makers to achieve a better comprehension of patients´ evaluation, which could be used to change and improve institutional behaviours to provide better PHC [48].

## Supporting information

S1 Questionnaire PMAQ Second cycle, Brazil, 2013–2014. (DOCX)

S1 Table Factors associated with patients’ satisfaction with dentists (n = 9,120) in primary health care, comparing scores up to 8 with scores of 9 or 10, using binary logistic regression, Brazil, 2013–2014. (DOCX)

S2 Table Factors associated with patients’ satisfaction with dentists (n = 9,120) in primary health care, comparing scores up to 9 with a score of 10, using binary logistic regression, Brazil, 2013–2014. (DOCX)
