# Supplementary material for: Factors associated with patients’ satisfaction in Brazilian dental primary health care
Source: PLoS One. 2017 Nov 16;12(11):e0187993. doi: 10.1371/journal.pone.0187993 (PMC5690593; doi:10.1371/journal.pone.0187993)
Supplement: S1 Questionnaire — (DOCX) [file pone.0187993.s001.docx]

| **Questionnaire PMAQ Second cycle, Brazil, 2013-2014** |
| --- |
| ***Questionário do segundo ciclo do PMAQ, Brasil, 2013-2014*** |
| **1 Sex** (Male, Female) |
| ***1 Sexo*** *(Masculino; Feminino)* |
| **2 How old are you?** (In years) |
| ***2 Qual é a idade do(a) senhor(a)?*** *(Em anos)* |
| **3 Do you work?** (Yes; No) |
| ***3 Você trabalha?*** *(Sim; Não)* |
| **4 What is your educational level?** |
| Illiterate |
| Read and write |
| From 1 to 7 years of education |
| 8 years of education |
| 9 to 10 years of education |
| 11 years of education |
| Incomplete college |
| Graduated college |
| Post-graduate |
| ***4 Até quando o(a) senhor(a) estudou?*** |
| *Não é alfabetizado (não sabe ler e escrever)* |
| *È alfabetizado (sabe ler e escrever)* |
| *Ensino fundamental incompleto* |
| *Ensino fundamental completo* |
| *Ensino médio incompleto* |
| *Ensino médio completo* |
| *Ensino superior incompleto* |
| *Ensino superior completo* |
| *Pós-graduação* |
| **5 Most of the time, how do you make your appointment with the dentist?** |
| By phone call |
| Using the internet |
| Personally visiting the Primary Health Care unit |
| Filling out a formal paper |
| Get in line to fill out formal paper |
| By the Community Health Agent? |
| ***5 Na maioria das vezes, como o senhor faz para marcar consulta com o dentista da sua equipe?*** |
| *Marca por telefone* |
| *Marca pela internet* |
| *Vai à unidade e marca o atendimento* |
| *Vai à unidade, mas tem que pegar ficha.* |
| *Vai à unidade e, para pegar ficha, é necessário fazer fila antes na unidade de saúde.* |
| *O agente comunitário de saúde marca a consulta* |
| **6 When given an appointment with the dentist, your appointment is:** |
| At a specific time |
| At a specific period of the day |
| In order of arrival |
| Trying to fit you, with no guarantee |
| Other way |
| ***6 Quando o(a) senhor(a) consegue marcar a consulta com o cirurgião dentista, sua consulta é:*** |
| *Com hora marcada* |
| *Em horários ou turnos definidos do dia* |
| *Por ordem de chegada* |
| *Encaixe* |
| *Outro(s)* |
| **7 Have you ever left the dental clinic with the next appointment scheduled?** (Yes; No) |
| ***7 O(a) senhor(a) já sai do atendimento com a próxima consulta agendada?*** *(Sim; Não)* |
| **8 Waiting time for dental appointment** (In days) |
| ***8 Tempo de espera*** *(Em dias)* |
| **9 In the clinic, how often were you guided by the oral health professionals about your health?** (Always; Almost always; Almost never; Never) |
| ***9 Nas consultas, com qual frequência o(a) senhor(a) é orientado(a) pelos profissionais de saúde bucal sobre os cuidados com a sua saúde buca****l****?****(Sempre; Na maioria das vezes; Quase nunca; Nunca)* |
| **10 During dental treatment, do the oral health professionals take notes in your dental records?** (Yes, always; Yes, sometimes; No) |
| ***10 Nas consultas, o(s) profissional (is) fazem anotações no prontuário ou ficha do (a) senhor(a)?****(Sim, sempre; Sim, algumas vezes; Não)* |
| **11 Do you think the time for dental treatment is enough?** (Yes, always; Yes, sometimes; No) |
| ***11 Durante o atendimento com os profissionais da equipe de saúde bucal, o senhor(a) acha que o tempo de consulta é suficiente?*** *(Sim, sempre; Sim, algumas vezes; Não)* |
| **12 When you looked for dental care without an appointment, did you receive care?** (Yes, always; Yes, sometimes; No) |
| ***12 Quando o senhor(a) procura o atendimento odontológico sem hora marcada é escutado?*** *(Sim, sempre; Sim, algumas vezes; Não)* |
| **13 What do you think about the way you were treated (or welcomed) when entering the oral health service?** (Very good; Good; Reasonable; Bad; Very bad) |
| ***13 O que o(a) senhor(a) acha sobre a forma como é recebido(a) ao procurar o serviço de saúde bucal?*** *(Muito bom; Bom; Razoável; Ruim; Muito Ruim)* |
| **14 Does the oral health information given to you in the clinic meet your needs?** (Yes, always; Yes, sometimes; No) |
| ***14 As orientações que os profissionais de saúde bucal dão para o(a) senhor(a) na unidade atendem às suas necessidades?*** *(Sim, sempre; Sim, algumas vezes; Não)* |
| **15 In general, do you think the facilities of the dental office are in good clean condition?** (Yes; No) |
| ***15 De forma geral, o que o(a) senhor(a) acha das instalações do consultório odontológico: Está em boas condições de limpeza*** *(Sim; Não)* |
| **16 In general, do you think the facilities of the dental office have good ventilation or air conditioning?** *(Yes; No)* |
| ***16* *De forma geral, o que o(a) senhor(a) acha das instalações do consultório odontológico:* Dispõe de boa ventilação ou climatização** *(Sim; Não)* |
| **17 In general, do you think the dental equipment is in good working condition?** *(Yes; No)* |
| ***17* *De forma geral, o que o(a) senhor(a) acha das instalações do consultório odontológico:*** ***Os equipamentos odontológicos estão em boas condições de uso*** *(Sim; Não)* |
| **18 In general, do you think the dental chair is in good working condition?** (Yes; No) |
| ***18* *De forma geral, o que o(a) senhor(a) acha das instalações do consultório odontológico:*** ***A cadeira odontológica está em boas condições de uso*** *(Sim, Não)* |
| **19 From 0 to 10, how would you grade your satisfaction with the treatment received from the dentist?** |
| ***19 De zero a dez, qual nota o (a) senhor(a) atribui para a sua satisfação com o atendimento recebido pelo(a) dentista?*** |
