# Supplementary material for: Factors associated with patients’ satisfaction in Brazilian dental primary health care
Source: PLoS One. 2017 Nov 16;12(11):e0187993. doi: 10.1371/journal.pone.0187993 (PMC5690593; doi:10.1371/journal.pone.0187993)
Supplement: S1 Table — (DOCX) [file pone.0187993.s002.docx]

**S1 Table Factors associated with patient’s satisfaction with dentist (n=9,120) in primary health care, comparing scores up to 8 with scores 9 or 10, using binary logistic regression, Brazil, 2013-2014**

| Variable | Unadjusted  Odds Ratio  (CI 95%) | P value | Adjusted  Odds Ratio  (CI 95%) | P Value |
| --- | --- | --- | --- | --- |
| 1. **Demographic Characteristics** | | | | |
| **Sex** | | | | |
| Male | 0.86 (0.75-1.00) | 0.044 | 0.73 (0.62-0.86) | <0.001 |
| Female | 1 |  | 1 |  |
| **Age** | 1.01 (1.01-1.02) | <0.001 |  |  |
| **Education level** | | | | |
| Post-graduate | 0.38 (0.20-0.74) | 0.004 | 0.35 (0.16-0.74) | 0.006 |
| Graduated College | 0.33 (0.20-0.52) | <0.001 | 0.27 (0.16-0.45) | <0.001 |
| Incomplete College | 0.34 (0.21-0.54) | <0.001 | 0.34 (0.20-0.57) | <0.001 |
| 11 years of education | 0.37 (0.25-0.54) | <0.001 | 0.35 (0.23-0.54) | <0.001 |
| 9 to 10 years of education | 0.41 (0.27-0.62) | <0.001 | 0.42 (0.27-0.65) | <0.001 |
| 8 years of education | 0.46 (0.30-0.69) | <0.001 | 0.47 (0.30-0.74) | 0.001 |
| From 1 to 7 years of education | 0.58 (0.39-0.86) | 0.007 | 0.60 (0.39-0.92) | 0.019 |
| Read and write | 0.66 (0.40-1.08) | 0.096 | 0.65 (0.38-1.13) | 0.125 |
| Illiterate | 1 |  | 1 |  |
| **Do you work?** | | | | |
| Yes | 0.71 (0.69-0.86) | <0.001 | 0.81 (0.71-0.92) | 0.001 |
| No | 1 |  |  |  |
| 1. **Access and Booking of Dental Appointments** | | | | |
| **Most of the time, do you make your appointment with the dentist by phone call?** | | | | |
| Yes | 0.96 (0.76-1.20) | 0.697 |  |  |
| No | 1 |  |  |  |
| **Most of the time, do you make your appointment with the dentist using internet?** | | | | |
| Yes | 0.57 (0.15-2.15) | 0.407 |  |  |
| No | 1 |  |  |  |
| **Most of the time, do you make your appointment personally visiting the PHC?** | | | | |
| Yes | 1.16 (1.03-1.30) | 0.012 |  |  |
| No | 1 |  |  |  |
| **Most of the time, do you make an appointment by filling a formal paper?** | | | | |
| Yes | 0.87 (0.76-1.00) | 0.041 |  |  |
| No | 1 |  |  |  |
| **Most of the time, do you need to get in line then fill formal papers to make the appointment?** | | | | |
| Yes | 0.75 (0.65-0.86) | <0.001 |  |  |
| No | 1 |  |  |  |
| **Most of the time, do you make your appointment with the dentist in the PHC by the Community Health Agent?** | | | | |
| Yes | 1.21 (1.05-1.39) | 0.010 |  |  |
| No | 1 |  |  |  |
| **When given an appointment with the dentist, your appointment is:** | | | | |
| Other way | 0.98 (0.48-2.03) | 0.960 |  |  |
| At a specific period of the day | 0.99 (0.88-1.11) | 0.876 |  |  |
| In order of arrival | 0.73 (0.53-0.99) | 0.040 |  |  |
| Trying to fit you, with no guarantee | 0.71 (0.32-1.57) | 0.398 |  |  |
| At a specific time | 1 |  | 1 |  |
| **Have you ever left the dental clinic with the next appointment scheduled?** | | | | |
| Yes | 1.49 (1.32-1.67) | <0.001 |  |  |
| No | 1 |  |  |  |
| **Waiting time for dental appointment** | | | | |
| Up to 7 days | 1.51 (1.36-1.69) | <0.001 | 1.16 (1.03-1.32) | 0.019 |
| 8 days or more | 1 |  | 1 |  |
| 1. **Bonding and Accountability** | | | | |
| **In the clinic, how often you were guided by the oral health professionals about your health?** | | | | |
| Never | 0.32 (0.28-0.37) | <0.001 | 0.45 (0.32-0.64) | <0.001 |
| Almost never | 0.13 (0.10-0.17) | <0.001 | 0.39 (0.28-0.54) | <0.001 |
| Almost always | 0.16 (0.12-0.22) | <0.001 | 0.62 (0.53-0.73) | <0.001 |
| Always | 1 |  | 1 |  |
| **During dental treatment, do the oral health professionals take notes in your dental records?** | | | | |
| No | 0.34 (0.25-0.47) | <0.001 |  |  |
| Yes, sometimes | 0.27 (0.21-0.34) | <0.001 |  |  |
| Yes, always | 1 |  |  |  |
| **Do you think the time for dental treatment is enough?** | | | | |
| No | 0.12 (0.18-0.25) | <0.001 | 0.32 (0.24-0.43) | <0.001 |
| Yes, sometimes | 0.21 (0.09-0.15) | <0.001 | 0.50 (0.41-0.60) | <0.001 |
| Yes, always | 1 |  | 1 |  |
| 1. **Welcoming of the Patient** | | | | |
| **When you look for the dental care without an appointment, did you received care?** | | | | |
| Yes, always | 2.20 (1.97-2.46) | <0.001 | 1.16 (1.01-1.32) | 0.034 |
| Sometimes/No | 1 |  | 1 |  |
| **What do you think about the way you were treated (or welcomed) when entered the oral health service?** | | | | |
| Very bad | 0.04 (0.02-0.09) | <0.001 | 0.13 (0.06-0.29) | <0.001 |
| Bad | 0.030 (0.02-0.05) | <0.001 | 0.11 (0.06-0.19) | <0.001 |
| Reasonable | 0.05 (0.04-0.06) | <0.001 | 0.10 (0.08-0.13) | <0.001 |
| Good | 0.25 (0.21-0.30) | <0.001 | 0.31 (0.26-0.38) | <0.001 |
| Very good | 1 |  | 1 |  |
| **Does the oral health information given to you in the clinic meet your needs?** | | | | |
| No | 0.08 (0.06-0.10) | <0.001 | 0.34 (0.24-0.48) | <0.001 |
| Yes, sometimes | 0.19 (0.16-0.22) | <0.001 | 0.47 (0.40-0.55) | <0.001 |
| Yes, always | 1 |  | 1 |  |
| 1. **Perception of Dental Facilities** | | | | |
| **In general, do you think the facilities of the dental office are in good clean condition?** | | | | |
| No | 0.27 (0.21-0.33) | <0.001 | 0.55 (0.42-0.72) | <0.001 |
| Yes | 1 |  | 1 |  |
| **In general, do you think the facilities of the dental office have good ventilation or air conditioning?** | | | | |
| No | 0.41 (0.35-0.48) | <0.001 | 0.74 (0.61-0.91) | 0.004 |
| Yes | 1 |  |  |  |
| **In general, do you think the dental equipment is in good working condition?** | | | | |
| No | 0.40 (0.35-0.46) | <0.001 |  |  |
| Yes | 1 |  |  |  |
| **In general, do you think the dental chair is in good working condition?** | | | | |
| No | 0.59 (0.52-0.68) | <0.001 | 0.78 (0.66-0.91) | 0.002 |
| Yes | 1 |  | 1 |  |
