# Supplementary material for: Factors associated with patients’ satisfaction in Brazilian dental primary health care
Source: PLoS One. 2017 Nov 16;12(11):e0187993. doi: 10.1371/journal.pone.0187993 (PMC5690593; doi:10.1371/journal.pone.0187993)
Supplement: S2 Table — (DOCX) [file pone.0187993.s003.docx]

**S2 Table Factors associated with patient’s satisfaction with dentist (n=9,120) in primary health care, comparing scores up to 9 with score 10, using binary logistic regression, Brazil, 2013-2014**

| Variable | Unadjusted  Odds Ratio  (CI 95%) | P value | Adjusted  Odds Ratio  (CI 95%) | P Value |
| --- | --- | --- | --- | --- |
| 1. **Demographic Characteristics** | | | | |
| **Sex** | | | | |
| Male | 0.85 (0.76-0.95) | 0.006 | 0.69 (0.60-0.79) |  |
| Female | 1 |  | 1 |  |
| **Age** | 1.02 (1.01-1.02) | <0.001 | 1.00 (1.00-1.01) | 0.035 |
| **Education level** | | | | |
| Post-graduate | 0.17 (0.10-0.30) | <0.001 | 0.13 (0.07-0.24) | <0.001 |
| Graduated College | 0.17 (0.11-0.25) | <0.001 | 0.13 (0.08-0.19) | <0.001 |
| Incomplete College | 0.22 (0.15-0.32) | <0.001 | 0.20 (0.13-0.30) | <0.001 |
| 11 years of education | 0.26 (0.19-0.35) | <0.001 | 0.24 (0.17-0.34) | <0.001 |
| 9 to 10 years of education | 0.30 (0.22-0.42) | <0.001 | 0.30 (0.21-0.43) | <0.001 |
| 8 years of education | 0.35 (0.25-0.49) | <0.001 | 0.34 (0.24-0.49) | <0.001 |
| From 1 to 7 years of education | 0.49 (0.36-0.68) | <0.001 | 0.50 (0.35-0.70) | <0.001 |
| Read and write | 0.64 (0.43-0.96) | 0.030 | 0.62 (0.40-0.95) | 0.029 |
| Illiterate | 1 |  | 1 |  |
| **Do you work?** | | | | |
| Yes | 0.73 (0.67-0.80) | <0.001 | 0.83 (0.75-0.92) | <0.001 |
| No | 1 |  | 1 |  |
| 1. **Access and Booking of Dental Appointments** | | | | |
| **Most of the time, do you make your appointment with the dentist by phone call?** | | | | |
| Yes | 0.99 (0.77-1.11) | 0.394 |  |  |
| No | 1 |  |  |  |
| **Most of the time, do you make your appointment with the dentist using internet?** | | | | |
| Yes | 1.35 (0.36-5.10) | 0.656 |  |  |
| No | 1 |  |  |  |
| **Most of the time, do you make your appointment personally visiting the PHC?** | | | | |
| Yes | 1.10 (1.01-1.21) | 0.036 |  |  |
| No | 1 |  |  |  |
| **Most of the time, do you make an appointment by filling a formal paper?** | | | | |
| Yes | 0.90 (0.81-1.01) | 0.069 |  |  |
| No | 1 |  |  |  |
| **Most of the time, do you need to get in line then fill formal papers to make the appointment?** | | | | |
| Yes | 0.88 (0.78-0.99) | 0.028 |  |  |
| No | 1 |  |  |  |
| **Most of the time, do you make your appointment with the dentist in the PHC by the Community Health Agent?** | | | | |
| Yes | 1.22 (1.09-1.37) | 0.001 |  |  |
| No | 1 |  |  |  |
| **When given an appointment with the dentist, your appointment is:** | | | | |
| Other way | 1.05 (0.58-1.89) | 0.870 | 1.50 (0.77-2.93) | 0.231 |
| At a specific period of the day | 1.08 (0.98-1.18) | 0.120 | 1.22 (1.10-1.36) | <0.001 |
| In order of arrival | 0.73 (0.56-0.95) | 0.017 | 0.94 (0.70-1.27) | 0.692 |
| Trying to fit you, with no guarantee | 0.79 (0.40-1.56) | 0.492 | 1.00 (0.45-2.23) | 0.996 |
| At a specific time | 1 |  | 1 |  |
| **Have you ever left the dental clinic with the next appointment scheduled?** | | | | |
| Yes | 1.37 (1.24-1.50) | <0.001 |  |  |
| No | 1 |  |  |  |
| **Waiting time for dental appointment** | | | | |
| Up to 7 days | 1.39 (1.27-1.52) | <0.001 | 1.13 (1.02-1.25) | 0.019 |
| 8 days or more | 1 |  | 1 |  |
| 1. **Bonding and Accountability** | | | | |
| **In the clinic, how often you were guided by the oral health professionals about your health?** | | | | |
| Never | 0.21 (0.16-0.28) | <0.001 | 0.46 (0.32-0.65) | <0.001 |
| Almost never | 0.16 (0.12-0.22) | <0.001 | 0.42 (0.30-0.58) | <0.001 |
| Almost always | 0.34 (0.30-0.38) | <0.001 | 0.56 (0.49-0.65) | <0.001 |
| Always | 1 |  | 1 |  |
| **During dental treatment, do the oral health professionals take notes in your dental records?** | | | | |
| No | 0.40 (0.29-0.54) | <0.001 |  |  |
| Yes, sometimes | 0.36 (0.29-0.47) | <0.001 |  |  |
| Yes, always | 1 |  |  |  |
| **Do you think the time for dental treatment is enough?** | | | | |
| No | 0.16 (0.12-0.21) | <0.001 | 0.40 (0.29-0.54) | <0.001 |
| Yes, sometimes | 0.27 (0.23-0.31) | <0.001 | 0.57 (0.47-0.68) | <0.001 |
| Yes, always | 1 |  | 1 |  |
| 1. **Welcoming of the Patient** | | | | |
| **When you look for the dental care without an appointment, did you received care?** | | | | |
| Yes, always | 1.97 (1.80-2.17) | <0.001 | 1.21 (1.09-1.36) | 0.001 |
| Sometimes/No | 1 |  | 1 |  |
| **What do you think about the way you were treated (or welcomed) when entered the oral health service?** | | | | |
| Very bad | 0.09 (0.04-0.20) | <0.001 | 0.20 (0.09-0.48) | <0.001 |
| Bad | 0.07 (0.04-0.11) | <0.001 | 0.20 (0.12-0.36) | <0.001 |
| Reasonable | 0.06 (0.05-0.08) | <0.001 | 0.12 (0.10-0.15) | <0.001 |
| Good | 0.29 (0.25-0.32) | <0.001 | 0.31 (0.28-0.36) | <0.001 |
| Very good | 1 |  | 1 |  |
| **Does the oral health information given to you in the clinic meet your needs?** | | | | |
| No | 0.10 (0.07-0.13) | <0.001 | 0.33 (0.22-0.48) | <0.001 |
| Yes, sometimes | 0.22 (0.20-0.25) | <0.001 | 0.49 (0.42-0.57) | <0.001 |
| Yes, always | 1 |  | 1 |  |
| 1. **Perception of Dental Facilities** | | | | |
| **In general, do you think the facilities of the dental office are in good clean condition?** | | | | |
| No | 2.61 (2.11-3.24) | <0.001 | 1.46 (1.12-1.90) | 0.005 |
| Yes | 1 |  | 1 |  |
| **In general, do you think the facilities of the dental office have good ventilation or air conditioning?** | | | | |
| No | 1.83 (1.58-2.13) | <0.001 |  |  |
| Yes | 1 |  |  |  |
| **In general, do you think the dental equipment is in good working condition?** | | | | |
| No | 2.08 (1.81-2.37) | <0.001 | 1.29 (1.10-1.52) | 0.002 |
| Yes | 1 |  | 1 |  |
| **In general, do you think the dental chair is in good working condition?** | | | | |
| No | 1.47 (1.30-1.65) | <0.001 |  |  |
| Yes | 1 |  | 1 |  |
